# A role for Kalirin-7 in nociceptive sensitization via activity-dependent modulation of spinal synapses

**DOI:** 10.1038/ncomms7820

**Published:** 2015-04-13

**Authors:** Jianning Lu, Ceng Luo, Kiran Kumar Bali, Rou-Gang Xie, Richard E. Mains, Betty A. Eipper, Rohini Kuner

**Affiliations:** 1 Department of Molecular Pharmacology, Pharmacology Institute, Medical Faculty Heidelberg, Heidelberg University, Im Neuenheimer Feld 366, 69120 Heidelberg, Germany; 2 Institute of Neuroscience, Fourth Military Medical University, 17 West Chang-le Road, Xi’an 710032, China; 3 Department of Neuroscience, University of Connecticut Health Center, 263 Farmington Avenue, Farmington, Connecticut 06030-3401, USA; 4 Department of Molecular Biology and Biophysics, University of Connecticut Health Center, 263 Farmington Avenue, Farmington, Connecticut 06030-3401, USA

## Abstract

Synaptic plasticity is the cornerstone of processes underlying persistent nociceptive activity-induced changes in normal nociceptive sensitivity. Kalirin-7 is a multifunctional guanine-nucleotide-exchange factor (GEF) for Rho GTPases that is characterized by its localization at excitatory synapses, interactions with glutamate receptors and its ability to dynamically modulate the neuronal cytoskeleton. Here we show that spinally expressed Kalirin-7 is required for persistent nociceptive activity-dependent synaptic long-term potentiation as well as activity-dependent remodelling of synaptic spines in the spinal dorsal horn, thereby orchestrating functional and structural plasticity during the course of inflammatory pain.

## Abstract (web summary)

The development of chronic inflammatory pain is linked with functional and structural modifications in nociceptive synapses. Here the authors demonstrate that Kalrinin-7 forms an essential link between synaptic receptors and cytoskeletal elements to functionally modify spinal nociceptive synapses.

## Graphical abstract

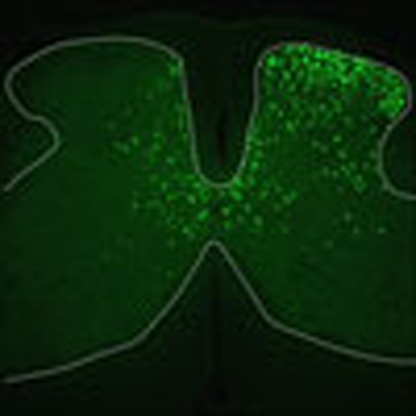

Activity-dependent changes in the structure and function of nociceptive pathways have been proposed to contribute to hyperalgesia^1^,^2^. Persistent nociceptive activity coming in from the periphery evokes synaptic potentiation at nociceptive synapses in the superficial spinal dorsal horn, with reports of pre- as well as post-synaptic contributions downstream of glutamatergic signalling^3^,^4^,^5^. In mouse models of pathological pain, an increase in the density of synaptic spines in the superficial spinal cord has been reported^6^,^7^. However, the proteins and pathways linking nociceptive activity-induced activation of synaptic glutamatergic receptors to structural and functional plasticity at these synapses are not well understood.

We hypothesized a role for synaptic proteins recruited by glutamatergic receptors that are capable of dynamically modulating the actin cytoskeleton. Synaptically expressed protein products of the *Kalrn* gene modulate the cytoskeleton locally by virtue of guanine nucleotide exchange factor (GEF) domains for key actin modulatory GTPases, such as Rac, RhoG and RhoA^8^,^9^.

The *Kalrn* gene encompasses 65 exons that can be spliced in various developmentally regulated patterns to yield several different proteins, such as the full-length proteins Kalirin-7 (Kal-7), Kalirin-9 (Kal-9) and Kalirin-12 (Kal-12), as well as their N-terminally truncated versions (Δ-Kal-7; Fig. 1a)^10^. The longest Kalirin isoform, Kalirin-12, includes an N-terminal Sec14-like domain, homologous to yeast Sec14p; nine spectrin repeats; Rac1/RhoG-GEF domain; SH3, Src homology 3 domain; RhoA-GEF domain; a second SH3 domain followed by a C-terminal Ser/Thr protein kinase domain^11^. Only full-length Kal-7 and its N-terminally truncated isoform, Δ-Kal-7, possess a C-terminal PDZ-interaction motif, encoded by the first 60 nucleotides of a 3′-terminal exon included by alternative splicing; this sequence promotes interactions with PDZ domain-containing proteins such as PSD95. Direct and indirect roles for Kal-7 in both NMDAR and AMPAR localization to synapses have been demonstrated^12^,^13^. Here we show that expression of specific splice variants of the *Kalrn* gene, particularly Kal-7, in spinal dorsal horn neurons contributes to inflammatory hypersensitivity via structure-function modulation of spinal synapses.

## Results

### Spinal expression of splice variants of the *Kalrn* gene

**Figure 1:**
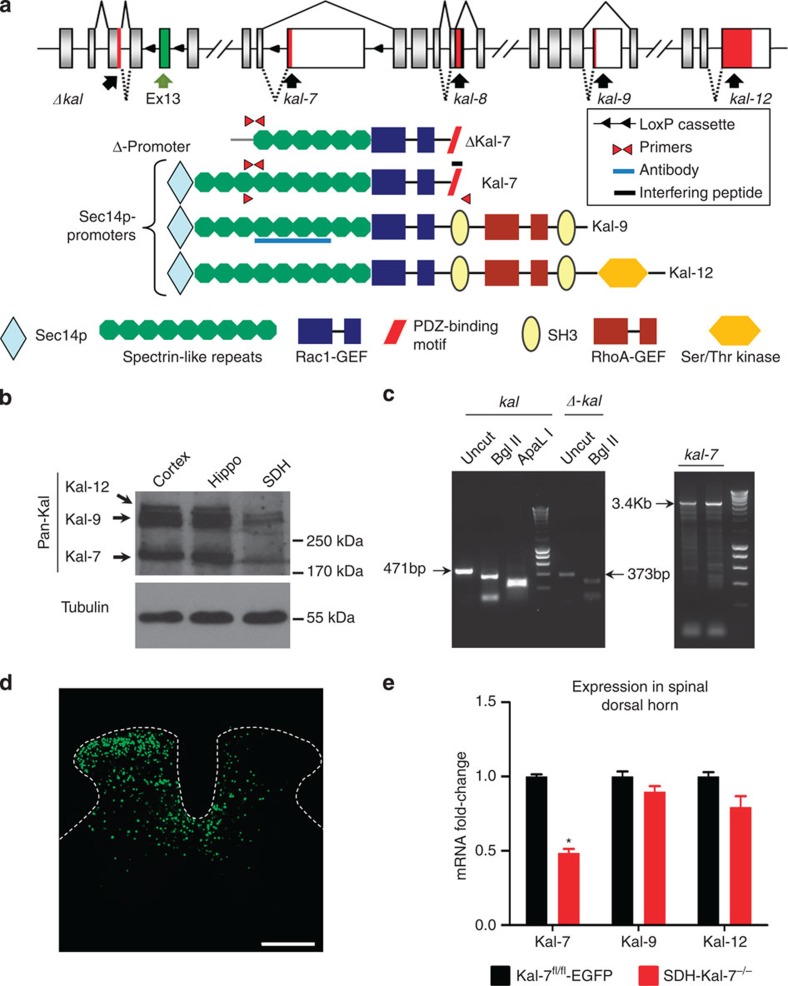
Expression of diverse Kalirins in the spinal dorsal horn and specific knockout of Kal-7 in spinal neurons. (**a**) Structure of the mouse *Kalrn* gene and the major protein products resulting via alternative splicing. Sites targeted by the reagents used in this study are shown. (**b**) Western blotting with antibody against the spectrin-repeat region detects expression of Kalirin in the spinal dorsal horn (SDH), with lysates from cortex and hippocampus as positive controls. (**c**) Transcripts for truncated or full-length Kalirins together with full-length Kalirin-7 (Kal-7) detected in the spinal dorsal horn via RT–PCR. (**d**) Unilateral microinjection of recombinant adeno-associated virus (rAAV) virus expressing Cre recombinase and EGFP into the mouse lumbar spinal dorsal horn (Scale bar, 300 μm). (**e**) Validation of Cre-mediated Kal-7 conditional knockout in mouse spinal dorsal horn (SDH-Kal-7^−/−^ mice) by quantitative RT–PCR, showing 50% reduction of Kal-7, but not Kal-9 or Kal-12 isoforms, in total lysate from spinal L4-L5 segments, as compared with AAV-EGFP-injected Kal-7^fl/fl^ mice (Kal-7^fl/fl^-EGFP control mice) (*n*=3 independent experiments). *P<0.05 as compared with control group; two-way ANOVA followed by Bonferroni *post-hoc* test. Error bars represent s.e.m.

All of the following analyses were oriented to the structure of *Kalrn* gene and splice variants shown schematically in Fig. 1a, which also depicts the key molecular tools used to study and manipulate expression of Kalirins in the spinal dorsal horn. Western blotting, performed using a previously validated pan-Kalirin antibody^14^ revealed the expression of all three major full-length Kalirin isoforms in the spinal cord (Fig. 1b; Supplementary Fig. 1 for full blot). Dense immunoreactivity for Kalirin proteins was observed in the neuropil of the spinal dorsal horn, which could be blocked by incubating with the corresponding peptide (Supplementary Fig. 2a).

To investigate the expression of distinct Kalirin isoforms in the mouse spinal cord, reverse transcription PCR (RT–PCR) analysis was carried out using primers spanning exon junctions (indicated in Fig. 1a; see details in Supplementary Tables 1 and 2). RT–PCR analysis on the spinal dorsal horn indicated the presence of N-terminally truncated and full-length Kalirin mRNAs, as well as the full-length mRNA specific to Kal-7; specificity of individual amplified DNA was examined by restriction digestion (Fig. 1c; Supplementary Fig. 2b). RT–PCR (Fig. 1c) as well as 3′ RACE (Supplementary Fig. 2c,d) further confirmed the expression of Kal-7 and Kal-12, which was verified by sequencing.

To investigate the role of products of the *Kalrn* gene, we utilized a conditional gene targeting approach using mice in which loxP sites were inserted in the genome to flank exon 13, which is commonly shared by all major protein isoforms that are generated via extensive alternative splicing^11^,^15^. To explore the selective contributions of Kal-7 versus other Kalirin isoforms, mice with loxP sites inserted in the genome to flank the exon specific to Kal-7 were used^16^. Recombinant chimeric adeno-associated virions (AAV) of serotypes 1 and 2 (AAV1/2) were used to unilaterally deliver Cre recombinase and EGFP selectively to the spinal dorsal horn (Fig. 1d and Supplementary Fig. 3a) to generate a spinal dorsal horn neuronal knockout of Kalirin (SDH-pan Kal^−/−^, or SDH-Kal-7^−/−^ mice). In previous studies, we have demonstrated neuronal tropism as well as a lack of toxicity of this AAV serotype on direct parenchymal injection into the spinal cord^7^,^17^.

Adult mice carrying floxed alleles of either Kalirin exon injected spinally with rAAV-EGFP virions served as controls (SDH-pan Kal^fl/fl^-EGFP or SDH-Kal-7^fl/fl^-EGFP). Quantitative RT–PCR analysis confirmed the specificity of the decrease in expression of Kal-7 versus other isoforms, such as Kal-9 and Kal-12, in the spinal cord of SDH-Kal-7^−/−^ mice as compared with SDH-Kal-7^fl/fl^-EGFP mice (Fig. 1e). Kal-7 expression dropped by 50% without a compensatory increase in other isoforms, such as Kal-9 or Kal-12, this observation is important because genetic deletion of Kal-7 in the mouse cortex is associated with a compensatory increase in cortical expression of Kal-9 and Kal-12 (ref. 16; Supplementary Fig. 3b).

### Functional impact of spinal Kalirin loss on pain

**Figure 2:**
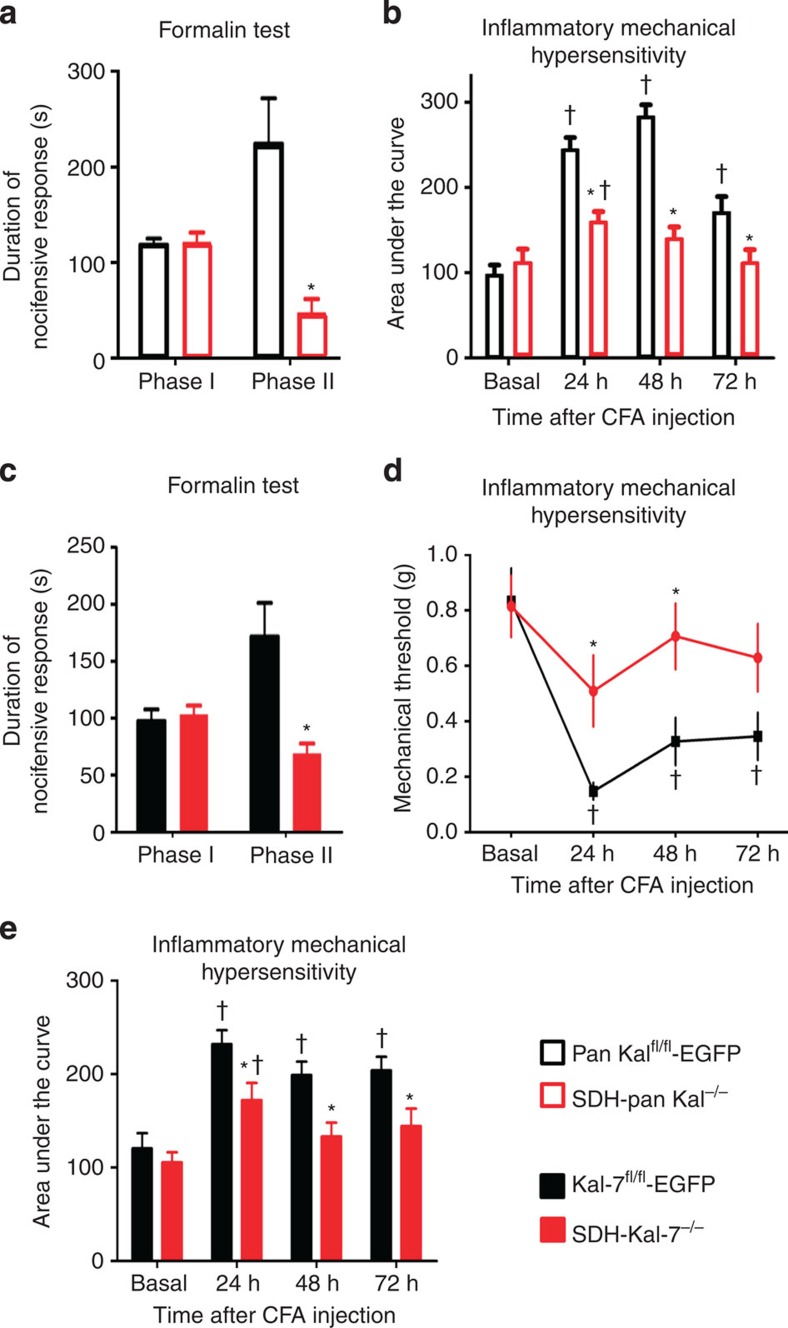
Functional impact of loss of all Kalirins or specifically Kal-7 in spinal dorsal horn neurons on pain-related behavior. (**a**) Suppression of Phase II, but not phase I, nocifensive behaviour induced by intraplantar formalin in mice lacking all Kalirins in spinal dorsal horn neurons (SDH-pan Kal^−/−^ mice) (*n*=7–8 per group). (**b**) CFA-induced mechanical hypersensitivity represented as area under the curve (AUC) of the entire von Frey stimulus-response curve over von Frey forces 0.04 to 1.4 g tested at various time points post-CFA injection in SDH-pan Kal^−/−^ mice as compared with control littermates (*n*=8–10 animals per group). (**c**) Suppression of Phase II nocifensive behaviour induced by intraplantar formalin in mice lacking Kal-7 specifically in spinal dorsal horn neurons (SDH-Kal-7^−/−^ mice) as compared with control littermates (*n*=8–9 per group). (**d**) Change in response threshold to mechanical force via von Frey filaments following intraplantar injection of complete Freund’s adjuvant (CFA) (*n*=10–11 animals per group). (**e**) CFA-induced mechanical hypersensitivity represented as area under the curve (AUC) of the entire von Frey stimulus-response curve over von Frey forces 0.04 to 1.4 g tested at various time points post-CFA injection (*n*=10–11 animals per group). In all panels, *P<0.05 as compared with control group; ^†^*P*<0.05 as compared with basal condition; two-way ANOVA followed by Bonferroni *post hoc* test. Error bars represent s.e.m.

In behavioural tests, acute pain, which develops within seconds and lasts for a few minutes following intraplantar injection of noxious chemicals, such as formalin (Fig. 2a), was unaffected by loss of all major Kalirin isoforms in the spinal dorsal horn from SDH-pan Kal^−/−^ mice; they showed comparable scratching, biting and paw flicking behaviours as control mice (Fig. 2a). Interestingly, however, formalin phase II behaviour, a form of short-term plasticity, which develops via central mechanisms as well as on-going activation of nociceptors^18^, was impaired following loss of spinal Kalirins (Fig. 2a).

To address spinal Kalirin contributions to more long-lasting nociceptive hypersensitivity developing due to persistent nociceptive activity, we utilized a model of unilateral hindpaw inflammation evoked by intraplantar injection of complete Freund’s adjuvant (CFA). Basal mechanical sensitivity to von Frey mechanical stimuli applied to the plantar surface of the paw was normal in SDH-pan Kal^−/−^ mice as compared with controls (Fig. 2b). When tested at 24, 48 and 72 h after CFA injection, SDH-pan Kal^−/−^ mice developed significantly less hypersensitivity to mechanical stimuli than SDH-pan Kal^fl/fl^-EGFP mice, as shown by an overall reduction in the CFA-induced increase in the integral of responses to plantar application of graded mechanical force (Fig. 2b).

We then focused on Kal-7, the most abundant isoform in adult CNS and the most interesting from the perspective of synaptic interactions^12^,^13^. SDH-Kal-7^−/−^ mice did not show any deviation from normal sensitivity to acute noxious stimuli, such as mechanical stimuli (von Frey hairs) applied to the paw plantar surface (Supplementary Fig. 4a). Furthermore, acute pain induced by intraplantar injection of formalin (phase I, Fig. 2c) was unaffected by loss of Kal-7 in the spinal dorsal horn. In contrast, formalin phase II behaviour was markedly impaired following loss of Kal-7 in the spinal dorsal horn (Fig. 2c). In the CFA model, when tested up to 72 h after CFA injection, SDH-Kal-7^−/−^ mice also developed significantly less hypersensitivity to mechanical stimuli than SDH-Kal-7^fl/fl^-EGFP mice, as evidenced by a significant attenuation of the CFA-induced drop in mechanical response thresholds (Fig. 2d) as well as overall reduction in the CFA-induced increase in the integral of responses to plantar application of graded mechanical force (Fig. 2e).

Thus, SDH-Kal-7^−/−^ mice fully recapitulated the nociceptive behavioral phenotype of SDH-pan Kal^−/−^ mice in terms of both nature and magnitude, indicating that spinal loss of Kal-7 can fully account for the deficits in early hypersensitivity and mechanical hyperalgesia/allodynia. Therefore, we focused on the unique functional attributes of Kal-7 in subsequent mechanistic experiments.

### Kal-7 modulates structural plasticity in spinal neurons

**Figure 3:**
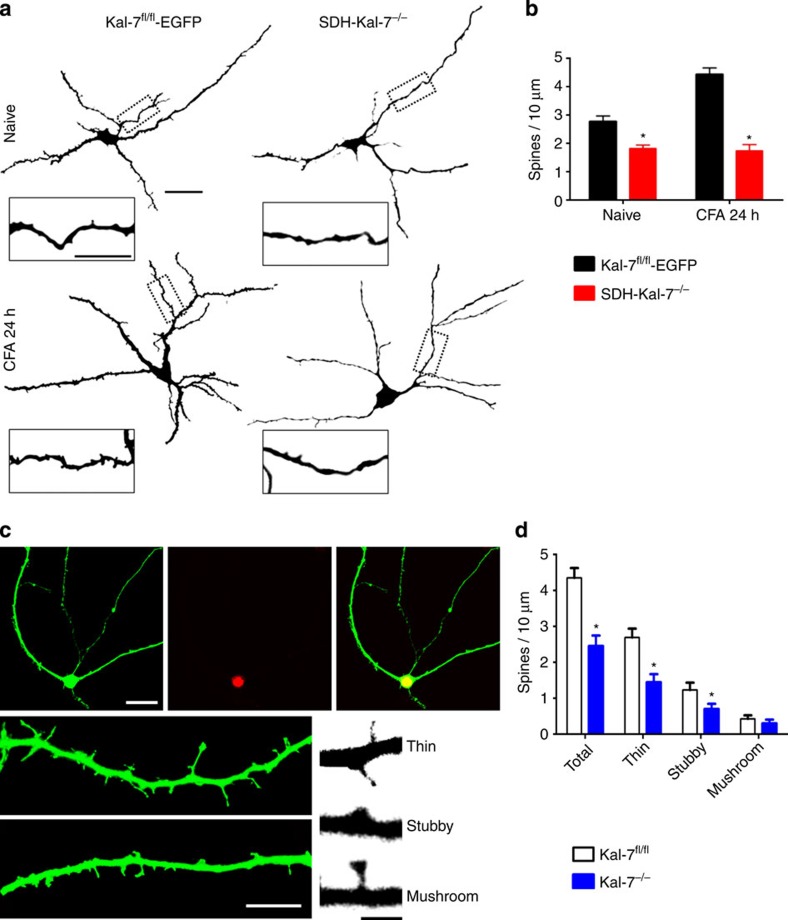
Role of Kal-7 signalling in dendritic spine remodelling in spinal dorsal horn neurons. (**a**) Typical examples of activity-dependent increase in dendritic spines following peripheral paw inflammation in control mice, but not in mice with spinal deletion of Kal-7 (overview scale bar, 50 μm, inset scale bar, 10 μm). (**b**) Quantification of spine density in Kal-7 knockout and control group under naive or CFA-treated condition *in vivo* (16–20 neurons were analysed from three animals each per treatment group). (**c**,**d**) Typical examples (**c**) and quantitative summary (**d**) of dendritic spines on specific deletion of Kal-7 in excitatory spinal cord neurons in culture via treatment with AAV-CKII-EGFP and AAV-Synapsin-Cre; double-positive neurons (merge) showed a lesser density of spines than Cre-negative neurons (scale bar, 50 μm in upper and 10 μm in lower panels); lower right, typical examples of different types of dendritic spines (scale bar, 1 μm). *P<0.05 as compared with control group; two-way ANOVA, Bonferroni *post hoc* test. Error bars represent s.e.m.

Synaptic localization and ability to activate Rho GTPases converge in Kal-7, allowing it to structurally modify the actin cytoskeleton at hippocampal synapses^16^. Here, we observed that Kal-7 expression in spinal dorsal horn neurons is required for nociceptive activity-induced structural plasticity. At 24 h following CFA-induced paw inflammation, Golgi-stained neurons in the superficial spinal laminae showed a significant increase in the density of dendritic spines on secondary and tertiary dendrites in control mice (examples in Fig. 3a and quantification in Fig. 3b), consistent with our previous findings^7^. Importantly, this increase in dendritic spine density on paw inflammation was suppressed in SDH-Kal-7^−/−^ mice (Fig. 3a,b). Moreover, spinal dorsal horn neurons in SDH-Kal-7^−/−^ mice showed a small, but significant, decrease in dendritic spine density in the basal state as compared with control mice (Fig. 3b).

As the nature of the neurons being analysed in the spinal cord *in vivo* cannot be easily identified via the Golgi technique, we employed cultures of spinal cord neurons from Kal-7^fl/fl^ mice; rAAV-CamKIIalpha-EGFP virions were used to identify dendritic spine-like structures on excitatory neurons and AAV-Cre virions were used to induce loss of Kal-7 (Fig. 3c). High-resolution fluorescence analysis on double-positive neurons enabled classifying synaptic spines into thin, stubby and mushroom subtypes, as described in detail under methods. Excitatory neurons lacking Kal-7 showed significantly fewer spines than excitatory neurons not infected with the Cre virions (control) within the same culture (Fig. 3d).

### Rac1 regulates spinal structural and functional plasticity

**Figure 4:**
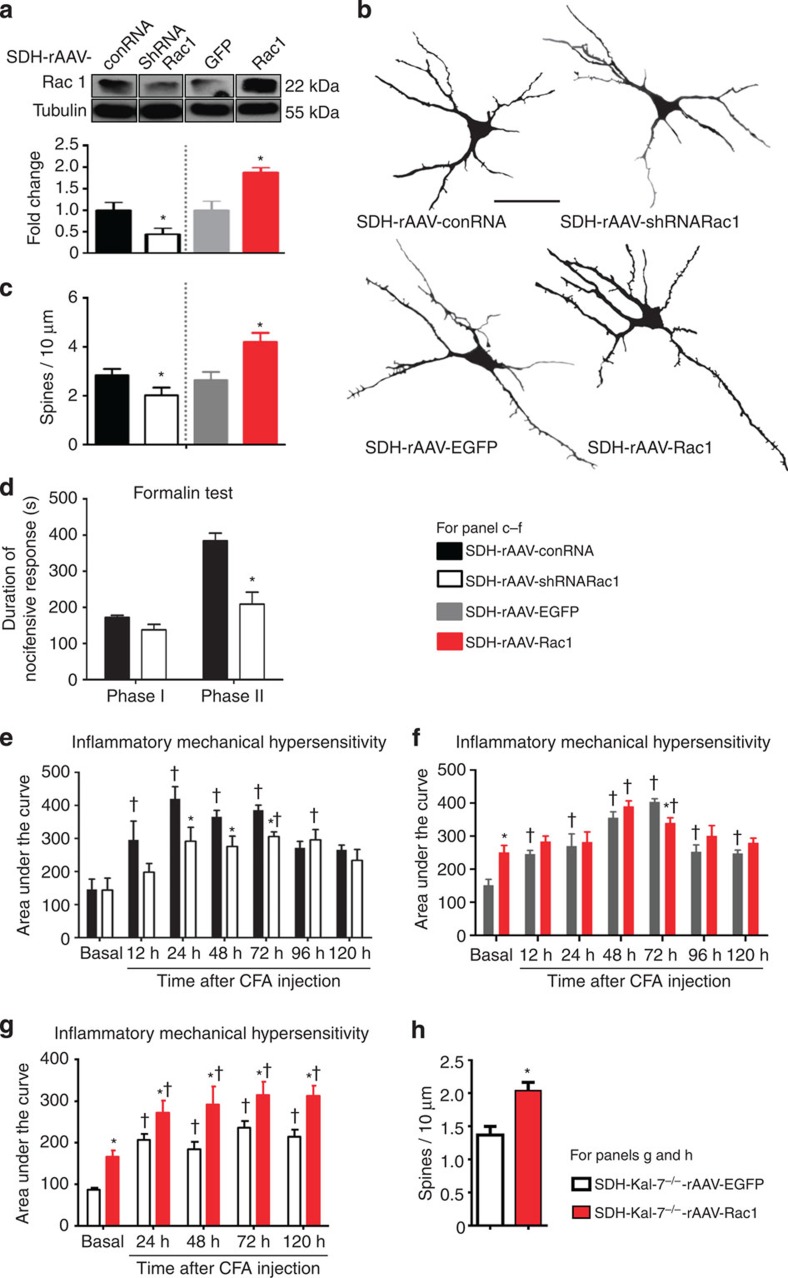
Bidirectional modulation of expression of Rac1 in spinal dorsal horn neurons *in vivo* and its structural and functional impact. (**a**) Western blotting of spinal lysates for validation of spinal knockdown and overexpression of Rac1 via AAV-mediated shRNA delivery or Rac1 cDNA delivery in comparison with corresponding controls. Lower panel represents quantitative summary of Rac1 expression normalized to corresponding Tubulin control. (**b**,**c**) Typical examples of traces derived from Golgi-impregnated spinal neurons (**b**) and their quantitative summary (**c**) in mice with spinal AAV-mediated knockdown or overexpression of Rac1, using mice spinally expressing a non-targeting shRNA or EGFP as controls (Scale bar, 50 μm; 15–18 neurons from three animals per treatment group). (**d**) Attenuation of phase II nocifensive behaviour, but not of phase I, in the formalin test by spinal knockdown of Rac1 (*n*=7–8 per group). (**e**) Spinal neuron-specific knockdown of Rac1 led to a reduction of mechanical hypersensitivity induced by intraplantar CFA injection as compared with mice expressing control RNA (*n*=6 per group). (**f**) Overexpression of Rac1 in neurons of the spinal dorsal horn led to enhanced basal sensitivity to mechanical nociceptive stimuli (*n*=6–8 mice). (**g**) Functional impact of overexpression of Rac1 or EGFP (control) in the spinal dorsal horn of SDH-Kal-7^−/−^ mice on sensitivity to mechanical nociceptive stimuli and CFA-induced hypersensitivity (*n*=6 mice per group). (**h**) Quantification of changes in spine density on overexpression of Rac1 or EGFP in spinal neurons lacking Kal-7 at 24 h post-CFA (25–30 neurons were analysed from from animals each per treatment group). *P<0.05 as compared with control group; ^†^*P*<0.05 as compared with basal condition; two-way ANOVA, Bonferroni *post hoc* test. Error bars represent s.e.m.

The structural effects of Kal-7 come about in part via its ability as a GEF to activate RhoG as well as Rac. Whereas RhoG is involved in axonal outgrowth^9^, Rac1 activation has been functionally linked to dendritic spine plasticity at hippocampal synapses^12^. Pharmacological blockade of Rac1 has also been reported to affect density of dendritic spines on spinal neurons in neuropathic pain states^19^,^20^. Here, we observed that knocking down the expression of Rac1 in spinal dorsal horn neurons, using the same *in vivo* viral strategy described above, diminished nociceptive sensitivity and spine density to the same extent as deletion of Kal-7. Selective knockdown of Rac1 in spinal dorsal horn neurons *in vivo* was achieved by spinally injecting AAV virions expressing a specific short hairpin RNA (SDH-rAAV-shRNARac1), with AAV virions expressing control RNA being employed as a control (SDH-rAAV-conRNA) (Fig. 4a; Rac1 expression was 65 +/− 9% of control). Neurons expressing Rac1 shRNA demonstrated decreased spine density compared with neurons expressing control RNA (see Fig. 4b for typical examples; Fig. 4c for quantitative summary).

*In vivo* behavioural analyses revealed that SDH-rAAV-shRNARac1 mice exhibited significantly reduced phase II formalin behaviour as compared with SDH-rAAV-conRNA mice (Fig. 4d), although the acute pain evident in Phase I (Fig. 4d) as well as basal responses to mechanical stimuli (Fig. 4e) was normal. Importantly, inflammatory mechanical hypersensitivity in the CFA model was diminished in SDH-rAAV-shRNARac1 mice as compared with SDH-rAAV-conRNA mice (summary of all von Frey filament strengths is shown in Fig. 4e).

To further address the relationship between spine density and nociceptive hypersensitivity, we then overexpressed Rac1 in the same neurons *in vivo* (SDH-rAAV-Rac1) using AAV virions expressing EGFP (SDH-rAAV-EGFP) as a control (Fig. 4a; Rac1 expression was enhanced by 55 +/− 14% over control), with the goal of increasing spine density in the spinal dorsal horn neurons targeted by our viral approach (Fig. 4b,c). Indeed, simulating activity-induced spine remodelling in the superficial spinal cord *in vivo* mimicked functional changes observed with peripheral inflammation: even in the basal state, SDH-rAAV-Rac1 mice showed enhanced mechanical sensitivity compared with control mice overexpressing EGFP (SDH-rAAV-EGFP) and their behaviour was not different from that of control mice following induction of paw inflammation (Fig. 4f).

### Interactions between Kal-7 and Rac1

It is well-established that Kal-7 can act as a RacGEF to activate Rac1 (refs 21, 22) and our above results revealed close parallels between their roles in nociceptive modulation as well as dendritic remodelling. However, since Kal-7 also has other signalling functions, for example, the ability to modulate the synaptic targeting of glutamate receptors^12^, it was of interest to address whether the above-described functions we observed for Kal-7 in spinal neurons are indeed mediated via Rac1. We therefore addressed whether Rac1 can rescue the phenotypic manifestations of a Kal-7 loss in spinal dorsal horn neurons. Interestingly, AAV-mediated overexpression of Rac1 in the SDH-Kal-7^−/−^ mice (SDH-Kal-7^−/−^-rAAV-Rac1) led to enhanced mechanical hypersensitivity compared with SDH-Kal-7^−/−^ mice overexpressing EGFP (SDH-Kal-7^−/−^-rAAV-GFP) in the basal state as well as following CFA-induced paw inflammation (Fig. 4g). Similarly, at 24 h following CFA-induced paw inflammation, Golgi-stained neurons in the superficial spinal laminae showed a significant increase in the density of dendritic spines on secondary and tertiary dendrites in SDH-Kal-7^−/−^ mice overexpressing Rac1 as compared with SDH-Kal-7^−/−^ mice overexpressing EGFP (Fig. 4h). Thus, Rac1 overexpression rescued the phenotypic deficits in SDH-Kal-7^−/−^ mice, indicating that, in spinal dorsal horn neurons, Kal-7 and Rac1 act in series.

### Kal-7 regulates long-term potentiation in spinal neurons

To further consolidate the role of Kal-7 in activity-dependent structure-function modulation of spinal nociceptive synapses, we sought to acutely disrupt the link between nociceptive input and postsynaptic Kal-7 recruitment in spinal neurons and analyse its impact on synaptic transmission and plasticity. We retrogradely labelled spinal dorsal horn neurons projecting to the periaqueductal grey (PAG) via injection of a fluorescent tracer (DiI) into the PAG *in vivo* and prepared acute lumbar spinal cord slices with attached dorsal roots 2-3 days thereafter, as described previously^3^. Patch clamp recordings were performed on DiI-labelled neurons and the attached dorsal roots were stimulated electrically at C-fibre strength to simulate nociceptive activity.

**Figure 5:**
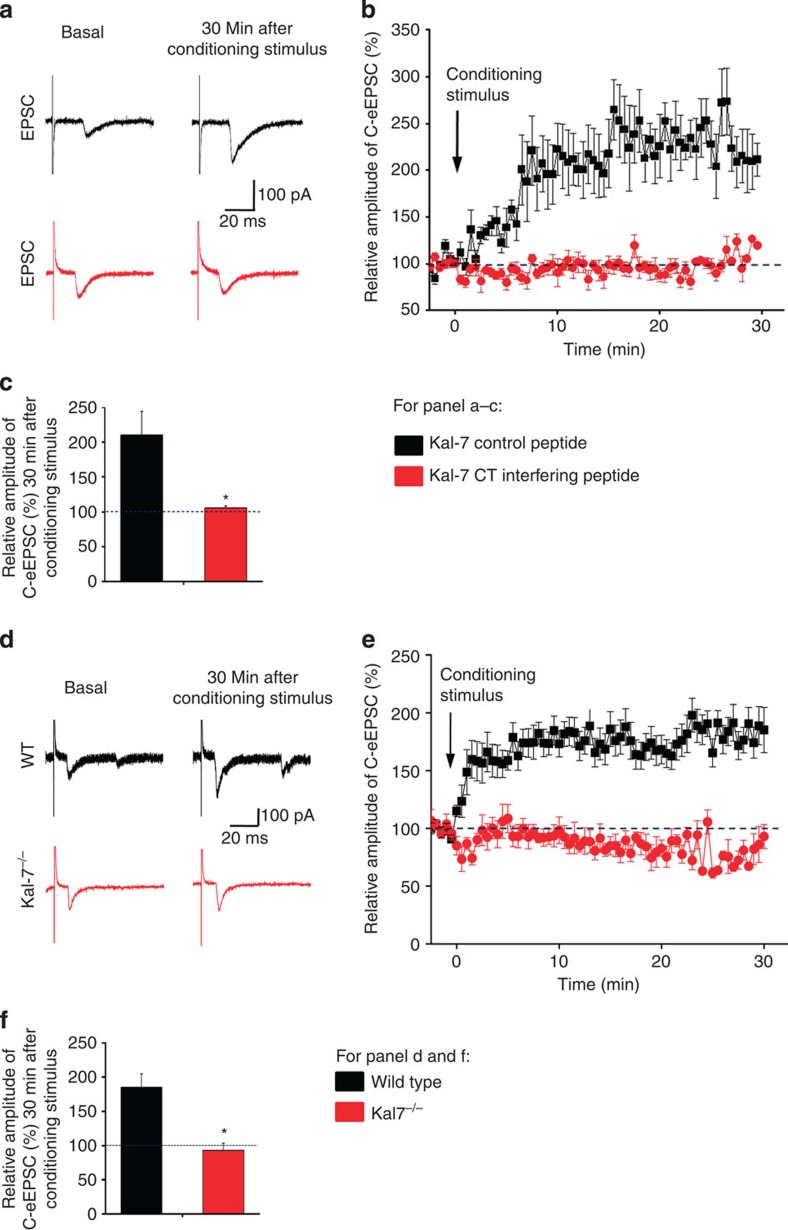
Functional impact of disrupting synaptic interactions of Kal-7 in spinal neurons or loss of Kal-7 expression on synaptic transmission. (**a**–**c**) At synapses between C nociceptors and spinal projection neurons, intracellular application of a Kal-7 C-terminal (CT) interfering peptide, but not a control peptide, blocked spinal long-term potentiation (*n*=8–10 slices). (**d**–**f**) Analysis of synaptic long-term potentiation in spinal neurons of mice lacking Kal-7 (Kal-7^−/−^ mice) and wild-type littermates (*n*=8–10 slices). Typical traces of evoked excitatory postsynaptic currents (eEPSCs) (**a**,**d**), time-course (**b**,**e**) and quantitative summary of % change in EPSC magnitude (**c**,**f**) are shown. *P<0.05 as compared with control group, Student’s *t*-test. Error bars represent s.e.m.

A Kal-7 C-terminal peptide previously characterized and demonstrated to disrupt synaptic protein–protein interactions of endogenously expressed Kal-7 (Kal-7 CT interfering peptide)^12^, or a corresponding control peptide with a mutation that renders it dysfunctional (Kal-7 control peptide), was included in the patch pipette solution. We observed that acutely disrupting synaptic Kal-7 interactions affected neither basal synaptic transmission between nociceptive C-fibres and spinal projection neurons nor influenced the membrane properties of spinal neurons (Supplementary Fig. 5). In spinal projection neurons, a conditioning stimulus of repetitive C-fibre stimulation (2 Hz, 2 min) applied to the dorsal roots induced synaptic long-term potentiation, as reported previously in rat^3^ and mouse^5^, which also came about when the Kal-7 control peptide was included in the patch pipette (Fig. 5a for typical traces of evoked EPSCs, Fig. 5b for time course and Fig. 5c for quantification of peak EPSCs). In contrast, when the Kal-7-CT interfering peptide was introduced via the patch pipette, the same nociceptive conditioning stimulus failed to induce LTP in spinal PAG projection neurons (Fig. 5a–c), and the evoked EPSCs were similar in magnitude to basal values. Thus, interfering with synaptic interactions of Kal-7 in spinal neurons inhibited the induction and expression of synaptic LTP.

To further consolidate these data, we also performed similar experiments in Kal-7 global knockout mice (Kal-7^−/−^ mice)^16^. Although the conditioning stimulus of repetitive C-fibre stimulation induced synaptic LTP in spinal neurons of wild-type littermates, Kal-7^−/−^ mice did not show LTP (see Fig. 5d for typical traces of evoked EPSCs, Fig. 5e for time course and Fig. 5f for quantification of peak EPSCs), further confirming a role for Kal-7 in spinal synaptic long-term potentiation.

## Discussion

Processes leading to synaptic potentiation are tantamount to enhancement of nociceptive sensitivity in states of nociceptive activity-dependent persistent pain^4^. This study now indicates a tight link between persistent nociceptive activity, structural plasticity at spinal dorsal horn synapses and functional expression of inflammatory mechanical hypersensitivity, which involves a key role for spinal Kal-7-Rac signalling.

Synaptic potentiation and central sensitization in spinal nociceptive networks are triggered by NMDAR activation and involve synaptic recruitment of AMPAR^1^,^2^,^4^,^23^. Our results indicate that a loss of Kal-7 in spinal neurons at spinal excitatory synapses impaired the induction of synaptic potentiation without altering basal synaptic transmission, the same manipulations *in vivo* attenuated inflammatory hypersensitivity without affecting basal nociception, thereby again highlighting the importance of synaptic plasticity in deviations from normal pain sensitivity. Mechanistically, these synaptic alterations are linked to protein–protein interactions between Kal-7 and proteins in the postsynaptic density, as a direct disruption of Kal-7 interactions with PDZ domain proteins via the Kal-7 CT peptide reproduced the loss of synaptic LTP.

The pleckstrin homology (PH) domain of Kal-7 can also directly interact with NR2B^13^. Indeed, a very recent study implicated Kalirin in mediating enhancement of the NR2B-PSD95 interaction and NR2B phosphorylation downstream of serum-inducible and glucocorticoid-inducible kinase 1 on spinal nerve ligation^24^. It should be noted, however, that, unlike neuropathic pain states, spinal NR2B has been reported to be downregulated in inflammatory pain models^25^,^26^. Taken together with our results, this suggests that Kalirin proteins share general functional similarities in spinal nociceptive sensitization with previously described, important synaptic glutamate receptor-interacting proteins such as PSD-95 and GRIP1, among others^27^.

However, our results also suggest that Kal-7 plays an additional, novel function in nociceptive sensitization that is distinct from previously described scaffolding proteins, by virtue of its ability to activate Rac1 at excitatory synapses. We observed that this enables dynamic alterations of dendritic spines in the spinal dorsal horn and induces long-term remodelling of nociceptive pathways. Spinal Rac1 activation has been implicated in several pathological pain states, such as diabetic neuropathic pain and spinal cord injury-evoked pain^6^,^20^, as well as inflammatory pain in this study. Whereas previous reports were based on the use of a pharmacological inhibitor, which may act globally on Rac1 across cell types, here we bidirectionally modulated Rac1 using molecular interventions that were specific to spinal dorsal horn neurons. Importantly, we observed a tight link between behaviourally assessed hypersensitivity and spine density when expression of Rac1 was modulated.

Exactly how nociceptive activity recruits cytoplasmic Rac1 in spinal neurons was unknown so far; our results indicate that, by virtue of its ability to function as a RacGEF^21^,^22^, Kal-7 can fulfil this function at spinal dorsal horn synapses. We observed that Rac1 overexpression could rescue behavioural defects in early and persistent inflammatory hypersensitivity as well as changes in spine density associated with a loss of Kal-7 in spinal neurons, indicating thereby that Rac1 acts downstream of Kal-7 at spinal synapses. Interestingly, the involvement of Kal-7 in multiple signalling pathways (ephrins, neuregulins and so on) and recent reports on its phosphorylation by multiple synaptically localized kinases identify it as a convergence point for synaptic modulation^28^. Taken together with our observations, this suggests that Kal-7 is well placed to orchestrate both functional and structural nociceptive plasticity in the spinal cord, thereby providing a basis for therapeutic modulation.

## Methods

### Animals

Age-matched 8- to 10-week-old wild-type C57BL6/j mice were purchased from Janvier (Le Genest Saint Isle, France) and maintained in an air-conditioned room with a light/dark (12/12) cycle. Transgenic lines were maintained by mating heterozygous mice. Fout to six littermates were fostered in a cage attached to the ventilation system (Tecniplast, Italy), with free access to food and water. All animal use procedures were in accordance with ethical guidelines imposed by the local governing body (Regierungspräsidium Karlsruhe, Germany).

Transgenic animals carrying the floxed allele for the Kal-7-specific exon of the *Kalrn* gene have been described previously. In brief, the targeting vector was designed to flank the Kalirin-7-specific exon with LoxP sites, as previously described^16^. Similar strategy was used disrupt the overall molecular function of Kalirins. Exon 13 of the *Kalrn* gene was targeted, on the premise that splicing of exon 12 to any of the subsequent 15 exons would lead to frame-shift mutation; the detailed gene targeting approach was described by ref. 11. To generate mice with conditional deletion in neurons of the spinal dorsal horn, Cre recombinase-expressing adeno-associated virions were delivered via microinjection directly into the parenchyma from transgenic animals carrying the either floxed alleles, as described in detail below.

### Total RNA isolation and reverse transcription

Total RNA was prepared from lumbar 3–5 segments of spinal dorsal horn tissue using the RNeasy Mini Kit (Qiagen, Hilden, Germany). First strand cDNA was synthesized from 1 μg of total RNA by using SuperScript III First-Strand Synthesis System (Invitrogen, Karlsruhe, Germany) by following manufacturer’s directions.

### RT–PCR and further validation

Exon spanning primers were designed to amplify the individual *Kalrn* mRNA transcripts based on the gene structure. PCR reactions were carried out in 50 μl, with 1 μl of diluted cDNA, 1 μl of forward and reverse primer (10 pmol per μl), 1 μl dNTP mix (10 mM), 5 μl of 10 × PCR reaction buffer (Takara, Japan), 1 μl of Long & Accurate (LA) Taq polymerase (5 U), and autoclaved ddH_2_O to 50 μl. The reaction program was as follows: 94 °C 2 min, 95 °C 10 s, 55–60 °C 30 s, 72 °C 30 s to 3.5 min, depending on the amplicon size, for 35 cycles, followed by final extension at 72 °C for 10 min. One-fifth volume of amplicons were checked over 1–1.5% agarose-TAE gel. Amplified DNAs of expected size were purified, digested with specific restriction enzymes or cloned into pGEM-T Easy vector (Promega, Wisconsin, USA) and sequenced for further verification.

For detecting the presence of mRNA from full-length Kalirin isoforms, a forward primer located in exon 9 and a reverse primer located in exon 11 were used. For visualization of the truncated isoforms, the forward primer was designed within the 5′ untranslated region (5′ UTR) of *ΔKal*, in the intron between exon 10 and exon 11; the reverse primer was in exon 12. The predicted size of the amplicons was calculated by running Primer-BLAST (www.ncbi.nlm.nih.gov/tools/primer-blast/) against the RefSeq mRNA database. The restriction enzyme site map for each amplicon was generated using NEBcutter V2.0 (tools.neb.com/NEBcutter2/); enzyme(s) were chosen and experimentally validated. For full-length Kal-7 detection, a forward primer in exon 9 and reverse primer in the Kal-7-specific exon were employed. Primers are listed in Supplementary Table 1.

### 3′ Rapid amplification of cDNA ends

3′ RACE was performed by using a 3′ RACE system for rapid amplification of cDNA ends (Invitrogen, Karlsruhe, Germany) using the manufacturer’s directions.

### Western blot

Fresh cerebral cortex, hippocampus or spinal cord tissues were quickly isolated from the animal after cervical dislocation. Tissues were weighed and immersed with 10 times amount of (v/w) lysis buffer containing 0.5% SDS, 50 mM Tris, pH 8.0, 1 mM DTT, 1 mM PMSF, 2 mM EDTA, 50 mM NaF, 0.2 mM sodium orthovanadate and proteinase inhibitor cocktail (Roche, Mannheim). Tissues were homogenized on a bench top homogenizer, sonicated and heated at 95 °C for 5 min. Supernatant was collected after centrifugation at 12,000 × *g*, RT for 10 min. Harvested protein (20–30 μg) was first separated on 6–8% separating gel, then transferred to a nitrocellulose membrane (Protran, 0.45 μm, Whatman, Dassel) at 250 mA for at least 1.5 h. Membrane with bound protein was blocked in 5% non-fat milk, in 1 × TBST for at least 30 min. Primary antibody JH2580 (pan-Kalirin, rabbit polyclonal, 1:1,000, generated in the laboratory of B. Eipper), anti-α-Tubulin (mouse monoclonal, T5168, 1:2,000, Sigma-Aldrich), anti-Rac1 (mouse monoclonal, 610651, 1:1,000, BD Bioscience) were diluted in blocking buffer and incubated with the membrane at 4 °C overnight. Membrane was further washed three times with 1 × TBST at room temperature for 15 min. Horseradish peroxidase (HRP)-conjugated secondary antibody (1:6,000) was diluted in blocking buffer and incubated with the membrane at room temperature for 1–2 h. Ultrasensitive ECL Plus chemiluminescent substrates (GE Healthcare) were used to visualize the signal, documented on an X-ray film (Fujifilm) and developed using a film processor. Membranes were stripped for 30 min at RT in an acidic stripping buffer and washed with 1 × TBST if re-probing with another antibody.

### Quantitative PCR analysis

Exon spanning primers were specially designed to amplify the mouse *Kalrn-7* or the housekeeping gene, *Gapdh* mRNA transcript. Twenty microlitres of reaction volume consisted of 5 μl of cDNA, 10 μl 2 × FastStart Essential DNA Green Master (Roche, Mannheim, Germany), 0.5 μl of forward and reverse primer (10 pmol per μl) and 4 μl of autoclaved ddH_2_O. The reaction program was as follows: 95 °C 10 min, 95 °C 10 s, 60 °C 10 s, 72 °C 10 s for 45 cycles, followed by melting curve analysis. Amplification and detection were carried out on a Roche LightCycler96 system (Roche, Mannheim, Germany); raw data were collected and further analysed on LightCycler96 Application Software. (For primer details please refer to Supplementary Table 2).

### Virus production and *in vivo* injection

pAAV-Syn-iCre2A-EGFP plasmid (harbouring chimera of AAV serotypes 1 and 2) was a generous gift from Dr Rolf Sprengel (Department of Molecular Neurobiology, Max Planck Institute for Medical Research, 69120 Heidelberg, Germany). A rAAV vector expressing GFP^7^ served as a control. shRNA against Rac1 (5′- ACC TCC TGA TAA GTT ACA GGT GCG TTT CAA GAG AAC GCA CCT GTA ACT TAT CAG TT -3′) or Rac1 cDNA was also subcloned into the rAAV backbone. Viral particles were produced and purified as we have previously described^29^,^30^. Spinal cord injections of virions (2–5 × 10^9^ particles per ml, 500 nl of a 2:1 mixture with 20% mannitol) in C57BL/6J adult mice were performed as previously described in detail^17^. Two tandem injections of AAV-Cre-2A-GFP virus achieved gene delivery spanning ∼4 mm without noticeable compromise along the mouse lumbar spinal dorsal horn. Neuronal tropism was achieved by expressing Cre recombinase under the control of the synapsin 1 promoter^31^; cell-type specificity was previously characterized^7^.

### Testing pain-related behaviour

All behavioural measurements were done in awake, unrestrained, age-matched male mice between 8 and 12 weeks old. For each time point, 4–6 animals from each group were involved, with the experimenter blind to the genotype. All tests were performed in an appropriately lighted, quiet room between 0900 and 1600 hours. Inflammation of the paw was induced by subcutaneous plantar injection of 20 μl complete Freund’s adjuvant (CFA, Sigma) under isoflurane anaesthesia, as described previously^32^. Behavioural testing was carried out in habituated mice by an observer blinded to the identity of the groups. Mechanical sensitivity was determined on paw withdrawal to manual application of graded von Frey hairs (0.04–1.4 g) to the plantar surface^32^. Formalin (1%, 20 μl) was injected into the plantar surface of the hindpaw, and the duration of nocifensive behaviours (lifting, licking or flinching) was measured over 60 min post injection^32^.

### Neuronal cultures and analysis of spine density

Primary spinal cord cultures derived from Kal-7^f/f^ transgenic embryos at embryonic day 15 were infected with rAAV-CaMKIIα-EGFP alone or together with rAAV-Syn-iCre2A-EGFP virions at 7 days *in vitro* (DIV), fixed at 28 DIV and examined with × 100 objective on a laser-scanning confocal microscope (Leica SP2 AOBS). Synaptic spines with typical morphology (thin, stubby or mushroom subtypes) were identified and counted based on previous description^33^.

To analyse dendritic spines *in vivo*, Golgi-Cox staining was performed using an FD Rapid GolgiStain Kit (FD Neurotechnologies, Ellicott, MD, USA) according to the protocol, as we have described in detail previously^7^. Bright-field stack images were captured on Nikon Eclipse Ni-E microscope (Nikon Imaging Center, University Heidelberg) with NIS Elements imaging software 4.0. Cells were traced using the Simple Neurite Tracer plug-in from ImageJ; dendritic spines were further analysed and quantified using ImageJ.

### Patch clamping

Lumbar spinal slices with attached dorsal roots were prepared from wild-type C57Bl/6j mice or from Kal-7^−/−^ mice stereotactically injected with DiI into the PAG; 2-3 days after DiI injection, spinal cord slices with dorsal roots attached were prepared and whole-cell patch clamp recordings were performed^5^. To induce synaptic potentiation, low-frequency stimulation (conditioning stimulus, 2 Hz for 2 min) was applied to the dorsal root. The dorsal root was stimulated with test pulses of 0.1 ms (intensity: 3 mA) to evoke EPSCs in the DiI-labelled neuron. In some experiments, Kalirin-7 interfering peptide, CT (10 μM) or mutant control peptide (10 μM) was intracellularly infused via a patch pipette. The recording mode remained the same as during the conditioning period. Recordings started 5–10 min after the formation of whole-cell configuration; interfering or control peptide was first dissolved in distilled water as 1,000-fold concentrated stock, and then diluted to the desired concentration in pipette solution immediately before use. Synaptic strength was measured by assessing the peak amplitudes of EPSCs. The mean amplitude of 4–5 EPSCs evoked by test stimuli before the conditioning stimulation was regarded as the control. Peak amplitudes of EPSCs every 30 s after conditioning were compared between two groups.

### Statistics

All data are expressed as mean±s.e.m. In behavioural, morphological and RT–PCR analyses, two-tailed unpaired Student’s *t*-test, one- or two-way ANOVA with *post-hoc* Bonferroni’s test was used. Differences with *P*<0.05 were considered to be significant.

## Additional information

**How to cite this article:** Lu, J. *et al*. A role for Kalirin-7 in nociceptive sensitization via activity-dependent modulation of spinal synapses. *Nat. Commun.* 6:6820 doi: 10.1038/ncomms7820 (2015).

## Supplementary Material

Supplementary Information Supplementary Figures 1-5 and Supplementary Tables 1-2
