# Supplementary material for: A role for Kalirin-7 in nociceptive sensitization via activity-dependent modulation of spinal synapses
Source: Nat Commun. 2015 Apr 13;6:6820. doi: 10.1038/ncomms7820 (PMC4403379; doi:10.1038/ncomms7820)
Supplement: Supplementary Information — Supplementary Figures 1-5 and Supplementary Tables 1-2 [file ncomms7820-s1.pdf]

a

Full blot for Kalirin expression analysis

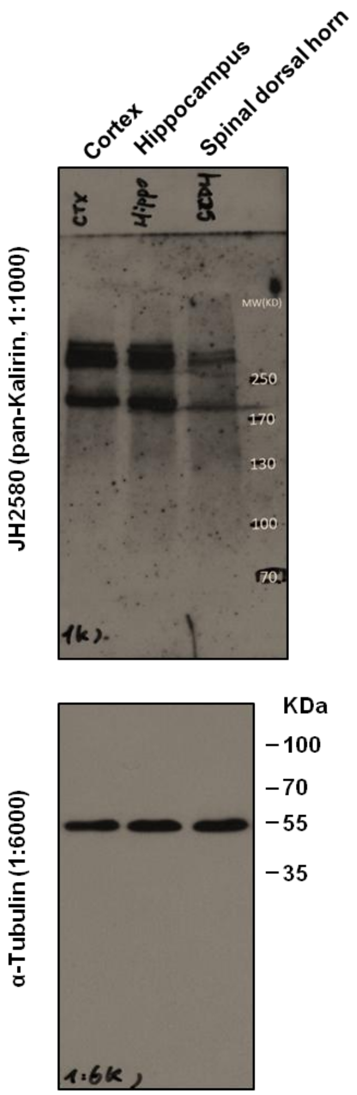

Supplementary Figure 1. Full blot for Kalirin expression analysis. Kalirin isoforms were detected with a pan antibody (JH2580), with Tubulin as loading controls.

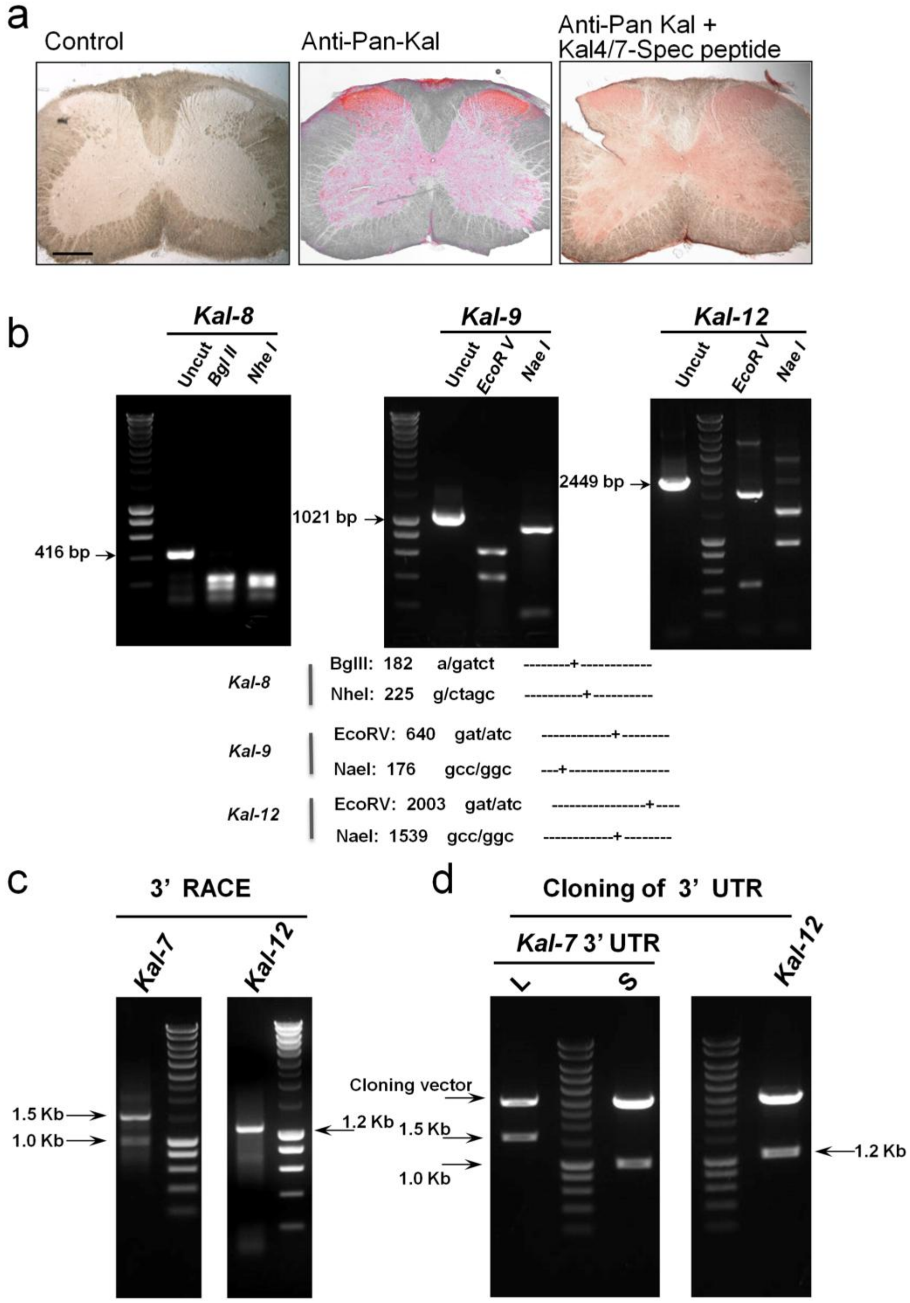

Supplementary Figure 2. Expression analysis of spinal dorsal horn Kalirin isoforms derived from the mouse *Kalrn* gene. (A) Immunohistochemistry with a pan-Kalirin antibody revealed immunoreactivity in the neuropil of the superficial spinal dorsal horn (middle), which was blocked by preincubation with the antigen-bearing peptide (far right) (Scale bar = 300 µm). (B) RT-PCR analysis of diverse Kalirin transcripts within the spinal cord. Individual amplicons were digested with specific restriction enzymes to verify specificity according to the site maps shown below. (C) 3' RACE was performed to validate the presence of functional full length isoforms of particular interest. Results showed that Kal-7 has two major versions of 3' UTR (1.0 Kb, 1.5 kb respectively). (D) Cloning of each individual 3' UTR (L - longer or S - shorter form in Kal-7).

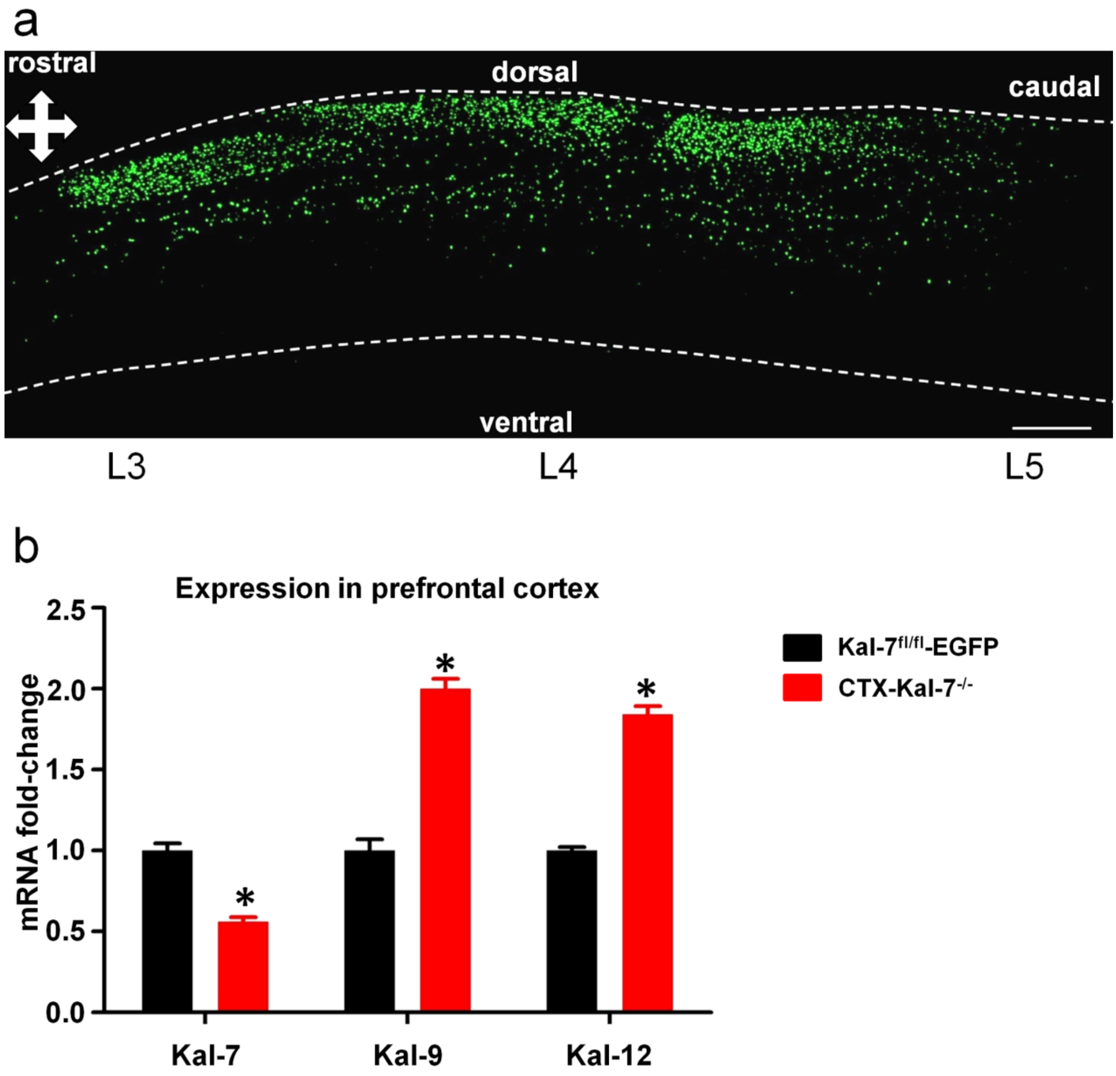

Supplementary Figure 3. Strategy for deletion of Kal-7 specifically in the spinal dorsal horn and analysis of other isoforms. (A) Two tandem injections AAV-Cre-2A-GFP into the spinal dorsal horn *in vivo* led to a rostro-caudally 4 mm long transduced area in the superficial lumbar spinal cord stretching over spinal segments L3, L4 & L5 (Scale bar = 300 µm). (B) Following AAV-Cre injection into the prefrontal cortex, Cre-mediated Kalirin-7 deletion was validated by quantitative RT-PCR. In contrast, expression of other major isoforms, such as Kal-9 and Kal-12, was increased (n = 3; * $p < 0.01$, data were collected from 3 independent experiments, two-way ANOVA followed by Bonferroni post-hoc test), as seen in other brain regions in the $Kal7^{-/-}$ mouse. Error bars represent standard error of the mean (s.e.m.).

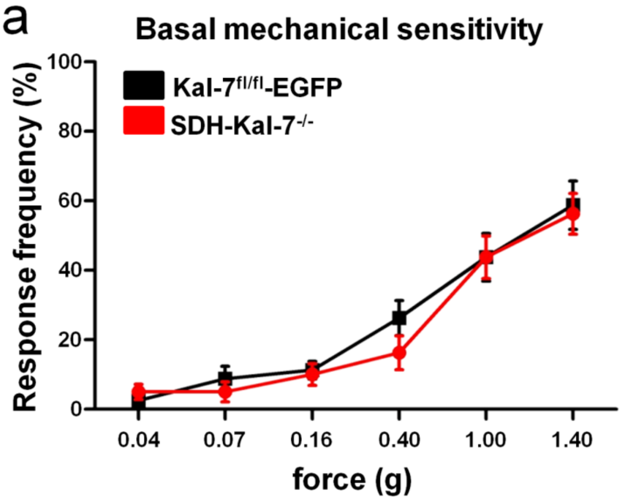

Supplementary Figure 4. Behavioral characterization of the effects of spinal deletion of Kal-7 via targeting distinct exons of the *Kalrn* gene. (A) Deletion of Kal-7 in spinal dorsal horn neurons (SDH-Kal7$^{-/-}$ mice) did not alter basal sensitivity to mechanical stimuli as compared to the control mice (n = 11-16 per group). Error bars represent standard error of the mean (s.e.m.).

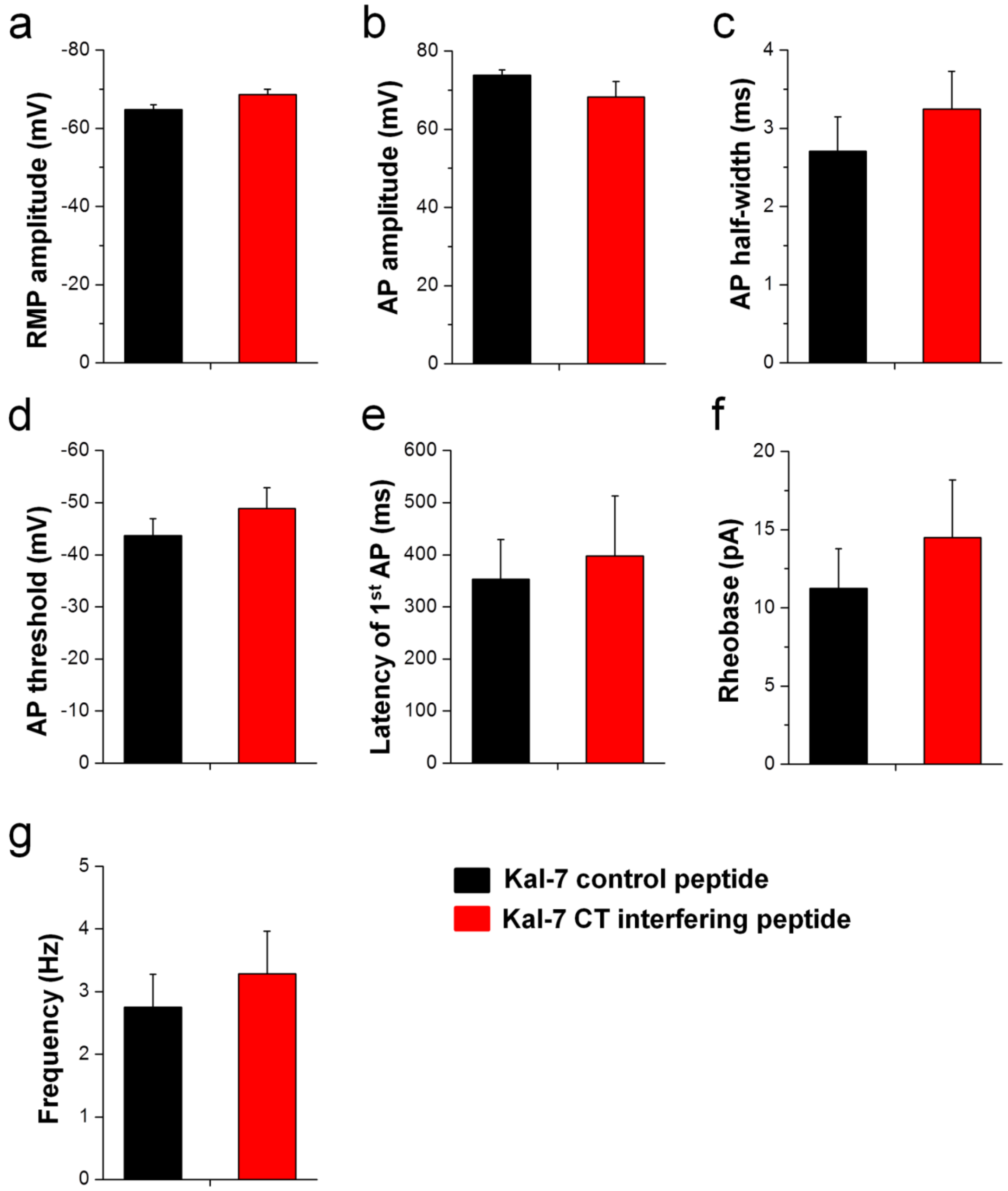

Supplementary Figure 5. Disrupting interactions of endogenous Kal-7 with PDZ domain proteins in spinal neurons via intracellular application of a Kal-7 C-terminal (CT) interfering peptide did not exert significant effect on membrane properties and excitability of spino-PAG projection neurons or basal synaptic transmission. Resting membrane potential (RMP), action potential (AP) properties, such as amplitude (A & B), half-width (C), threshold (D), latency of first AP (E), Rheobase (F) and firing frequency (G) to depolarizing step current were not altered by Kal-CT interfering peptide (10 μM). Error bars represent standard error of the mean (s.e.m.).

Supplementary Tables:

Supplementary Table 1 Primers for RT-PCR

Gene Name	Oligo Name	Sequence (5'->3')
Full front Kalirin	Kal-Top-FL	CACAGCCACAGCCAACTACTCCAAG
	Kal-FLI-3'	TTGGTGTGCGTGTGGAAAGAGAC
Delta front Kalirin	ΔKal-Top	TGACTTTGCCTCACTTTTGTTGCTG
	Kal-Δ-3'	TCTTCGCCTTCCTTGATGACATT
Kalirin7-specific	KalEx33A_for	ATGGGGTAGAGGATGGTGACAG
	Kal7_UTR_rev2	AGGTTGGGAGCTGGGGAAGTAC
Kalirin8-specific	KalEx36A_for	GACAACGACCCCACACAGGATG
	Kal8_UTR_rev	TCGTGAAATTAGGATGTGGGAG
Kalirin9-specific	KalEx48A_for	CTCTGCCACCCCTGAAGATAT
	Kal9_UTR_rev	AAATGTAGCAACGGAAGGCATG
Kalirin12-specific	KalEx59A_for	GCTGATGGAAGAAAAGGTAGCT
	Kal12_UTR_rev	CTTGGAGTAGCTGCCGTTGTGG

Supplementary Table 2 Primers for Quantitative Real Time-PCR

Gene Name	Oligo Name	Sequence	Tm (℃)	Expected size
GAPDH NM_008084.2	mrGapdh-top	TTGTCAGCAATGCATCCTGCACCACC	61	119 bp
	mrGapdh-rev2bot	CTGAGTGGCAGTGATGGCATGGAC	61	
Kalirin7-specific	KalEx33-for	GATACCATATCCATTGCCTCCAGGACC	61	127 bp
	Kal7unique-rev	CCAGGCTGCGCGCTAAACGTAAG	62	
Kalirin9-specific	KalEx51-for	GCCCCTCGCCAAAGCCACAGC	62	125 bp
	Kal9unique-rev	CCAGTGAGTCCCGTGGTGGGC	62	
Kalirin12-specific	KalEx62-for	CAGCAGCCACGTGCCTGCAGC	62	140 bp
	KalEx62-rev	TCTTGACATTGGGAATGGGCCGCAC	61	
